# Supplementary material for: Spatial and temporal tracking of cardiac exosomes in mouse using a nano-luciferase-CD63 fusion protein
Source: Commun Biol. 2020 Mar 10;3:114. doi: 10.1038/s42003-020-0830-7 (PMC7064570; doi:10.1038/s42003-020-0830-7)
Supplement: Supplementary file 1 — Supplementary figures and methods [file 42003_2020_830_MOESM1_ESM.pdf]

Supplementary Figures

Supplement Figure 1.

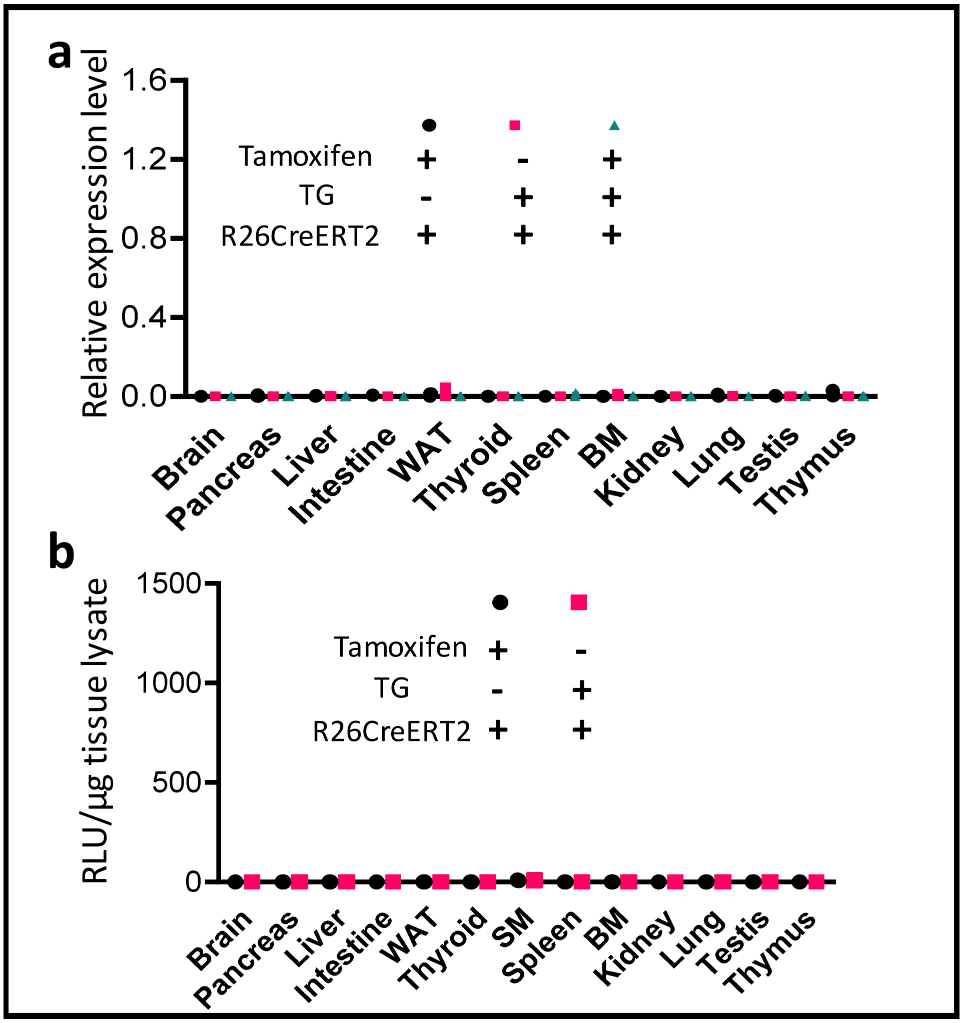

Supplement Fig. 1. CD63NanoLuc expression leakage was minimal. a. CD63NanoLuc transcripts in non-cardiac tissues were not detected by qPCR. n=4. **b.** CD63NanoLuc luciferase activities were not exhibited without tamoxifen induction in R26CreERT2; TG-αMHC-STOP-CD63NanoLuc mice. TG: TG-αMHC-STOP-CD63NanoLuc, n=4. All bar graph expressed as mean ± SD.

Supplement Figure 2.

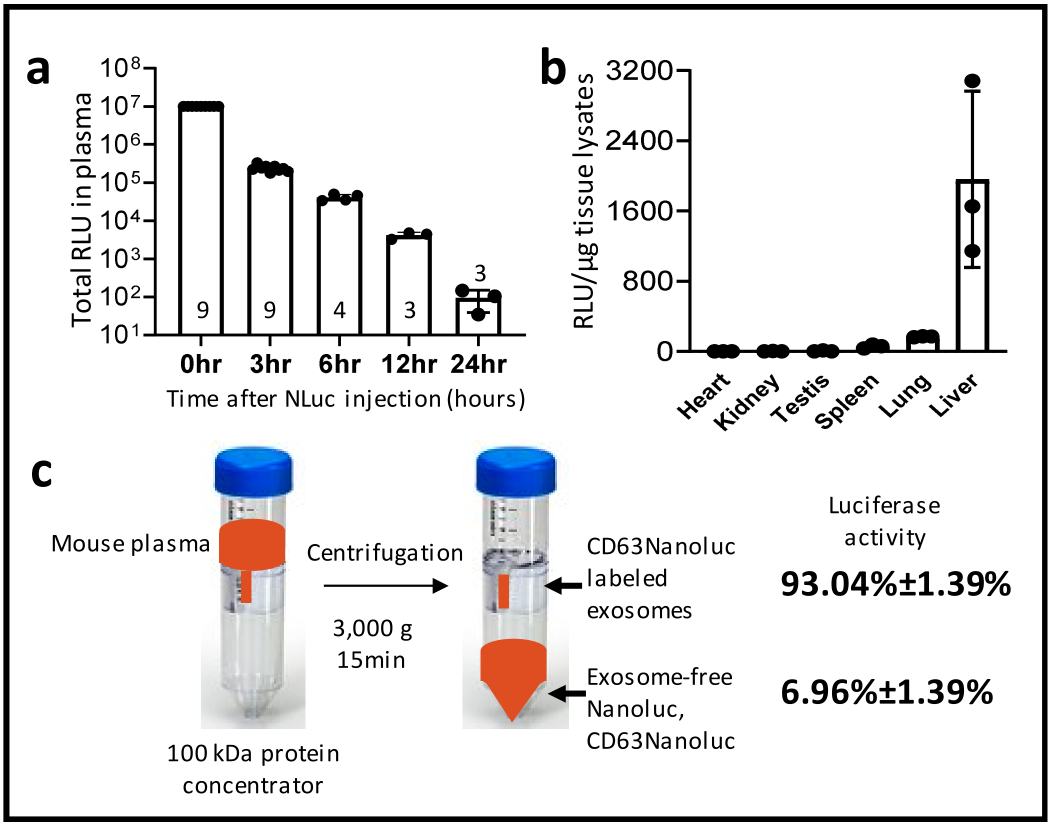

Supplement Fig. 2. Free NanoLuc protein degraded rapidly in mouse plasma and mainly deposited to liver. a. Time course of the total bioluminescence signal in mouse plasma after NanoLuc tail vein injection. Sample sizes are indicated on each column. $p<0.0001$. **b.** Tissue uptakes of NanoLuc protein were shown by luciferase activity, $p=0.0004$, $n=3$. **c.** Exosome-free NanoLuc molecules were separated from exosome conjugated NanoLuc by 100kDa protein concentrator. Luciferase activities in exosome-free NanoLuc and exosome-associated NanoLuc were assessed by luciferase assay, $n=9$. All bar graph expressed as mean ± SD.

Supplement Figure 3.

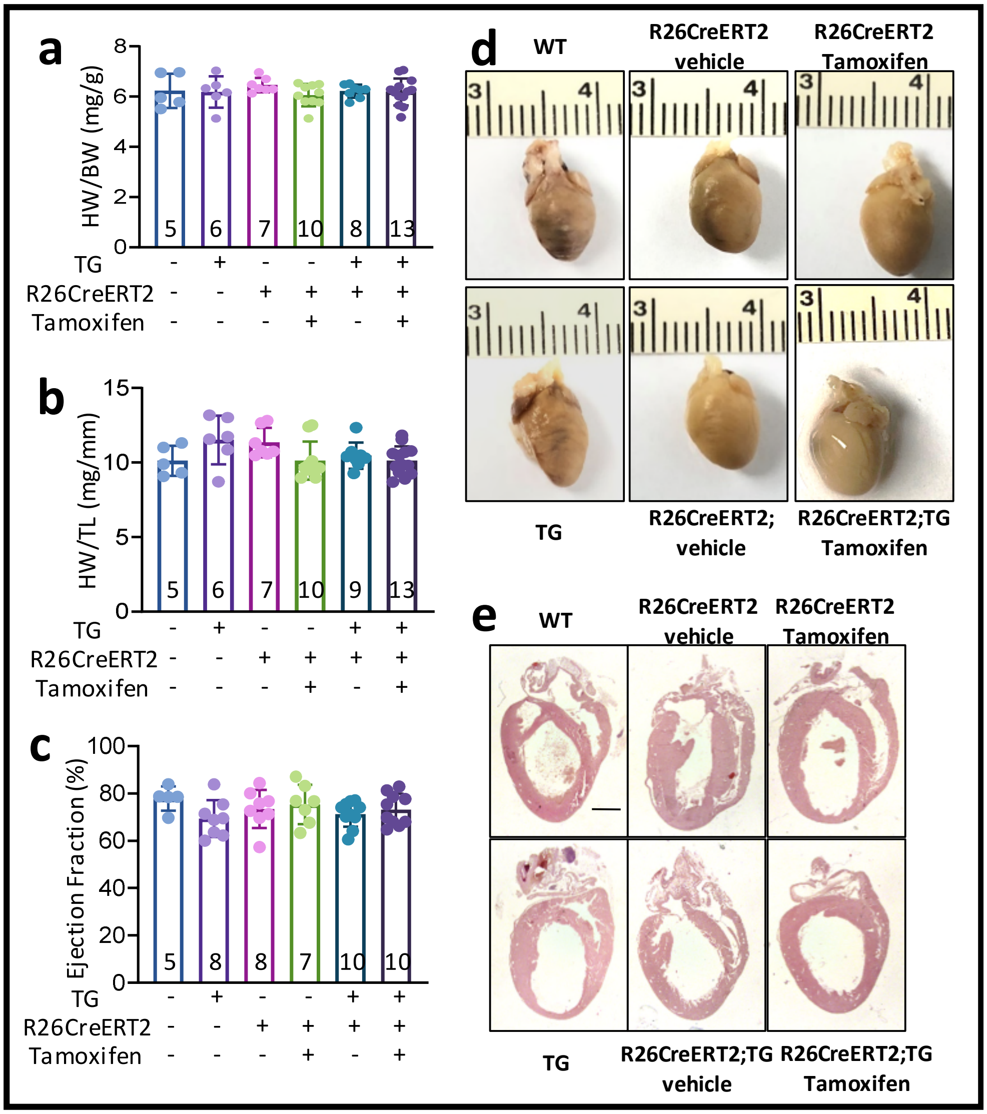

Supplement Fig. 3. Cardiac assessment of CD63NanoLuc expression in the mice. Cardiac morphology (**a-b, d**), histology by hematoxylin and eosin staining (**e**) and cardiac function by echocardiography (**c**) were analyzed. Scale bar: 1mm. The numbers in the columns represented the number of animals in each group. TG: TG-αMHC-STOP-CD63NanoLuc. Sample sizes are indicated on each column. All bar graph expressed as mean ± SD.

Supplement

Figure 4.

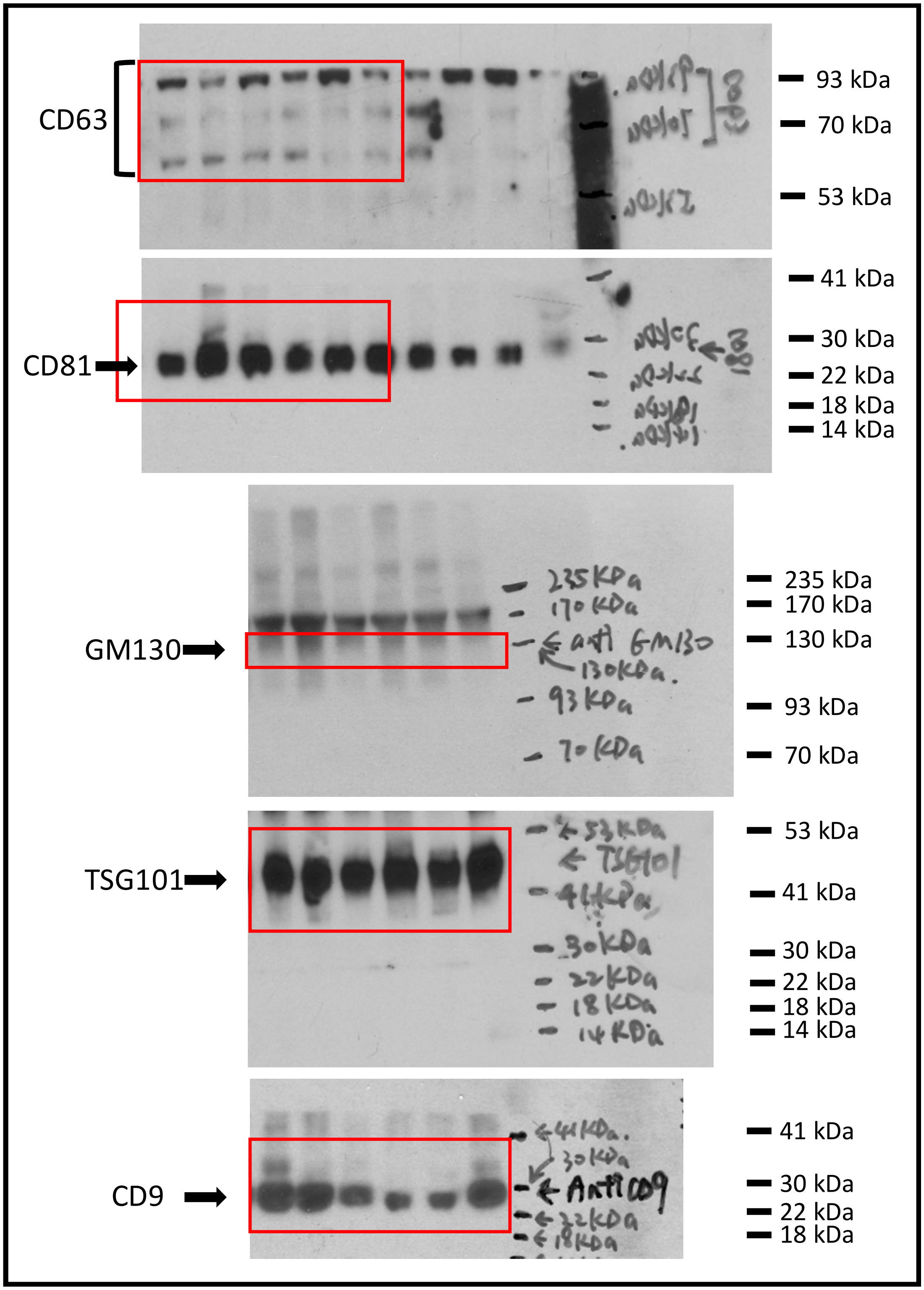

Supplement Fig. 4. Western blot gel full images.

Supplementary Methods:

NanoLuc protein expression and delivery: NanoLuc expression vector fused with His tag was constructed to pcDNA3.1(-)A . The expression of NanoLuc was achieved by transient transfection in 293 cells using Lipofectamine 3000 (L3000015, ThermoFisher Scientific, MA, USA). 293 cell lysate was collected 48 hours after transfection. NanoLuc protein was purified from the cell lysate by Ni-MagBeads (L00327, GenScript, Piscataway, NJ, USA) following manufacture instruction. $1X10^7$ RLU of NanoLuc protein were delivered to the mice circulation by tail vein injection. Mouse plasma samples were collected 3, 6, 12 and 24 hours after injection. Tissue samples from mice were collected 12 hours after NanoLuc protein injection.

Free NanoLuc separation from the plasma: To separate free NanoLuc with exosome-associated NanoLuc , plasma exosomes were filtered through the 100,000Da protein concentrator (88503, ThermoFisher Scientific, MA, USA) by centrifugation at 15,000g for 10 minutes at 4˚C. The flowthrough, which contains the exosome free NanoLuc, was subjected to luciferase assay.

Cardiac function assessment: Cardiac function was assessed by Vevo770 High-Resolution Micro-Imaging System (VisualSonics, Toronto, ON, Canada) as described previously[1]

Histology: Mouse whole heart were dissected and sectioned as described previously[1]. The animal body weight, heart weight and tibia length were quantified. Whole heart paraffin sections were subjected to hematoxylin & eosin staining as described previously[1]. Pictures were taken under the 2.5X microscope objective

Supplementary References:

1. Yang, X. et al. Genetic deletion of Rnd3/RhoE results in mouse heart calcium leakage through upregulation of protein kinase A signaling. *Circ Res* **116**, e1-e10 (2015).
