# Supplementary material for: Spatial and temporal tracking of cardiac exosomes in mouse using a nano-luciferase-CD63 fusion protein
Source: Commun Biol. 2020 Mar 10;3:114. doi: 10.1038/s42003-020-0830-7 (PMC7064570; doi:10.1038/s42003-020-0830-7)
Supplement: Supplementary file 2 — Descriptions of additional supplementary files [file 42003_2020_830_MOESM2_ESM.pdf]

Description of additional supplementary files

Supplementary Data 1: Source Data for Figure 2.

Supplementary Data 2: Source Data for Figure 4.

Supplementary Data 3: Source Data for Figure 5.

Supplementary Data 4: Source Data for Supplementary Figure 1.

Supplementary Data 5: Source Data for Supplementary Figure 2.

Supplementary Data 6: Source Data for Supplementary Figure 3.
