# Supplementary material for: Spatial and temporal tracking of cardiac exosomes in mouse using a nano-luciferase-CD63 fusion protein
Source: Commun Biol. 2020 Mar 10;3:114. doi: 10.1038/s42003-020-0830-7 (PMC7064570; doi:10.1038/s42003-020-0830-7)
Supplement: Supplementary file 10 — Reporting Summary [file 42003_2020_830_MOESM10_ESM.pdf]

nature research

Corresponding author(s): Jiang Chang

Last updated by author(s): Jan 15, 2020

Reporting Summary

Nature Research wishes to improve the reproducibility of the work that we publish. This form provides structure for consistency and transparency in reporting. For further information on Nature Research policies, see Authors & Referees and the Editorial Policy Checklist.

Statistics

For all statistical analyses, confirm that the following items are present in the figure legend, table legend, main text, or Methods section.

n/a	Confirmed	
☐	☒	The exact sample size (*n*) for each experimental group/condition, given as a discrete number and unit of measurement
☐	☒	A statement on whether measurements were taken from distinct samples or whether the same sample was measured repeatedly
☐	☒	The statistical test(s) used AND whether they are one- or two-sided<br>*Only common tests should be described solely by name; describe more complex techniques in the Methods section.*
☒	☐	A description of all covariates tested
☐	☒	A description of any assumptions or corrections, such as tests of normality and adjustment for multiple comparisons
☐	☒	A full description of the statistical parameters including central tendency (e.g. means) or other basic estimates (e.g. regression coefficient) AND variation (e.g. standard deviation) or associated estimates of uncertainty (e.g. confidence intervals)
☐	☒	For null hypothesis testing, the test statistic (e.g. F, t, r) with confidence intervals, effect sizes, degrees of freedom and P value noted<br>*Give P values as exact values whenever suitable.*
☒	☐	For Bayesian analysis, information on the choice of priors and Markov chain Monte Carlo settings
☒	☐	For hierarchical and complex designs, identification of the appropriate level for tests and full reporting of outcomes
☒	☐	Estimates of effect sizes (e.g. Cohen's d, Pearson's r), indicating how they were calculated

Our web collection on statistics for biologists contains articles on many of the points above.

Software and code

Policy information about availability of computer code

Data collection	Relative Luciferase unit was recorded by using ICE software V1.0.9.0 (Berthold).<br>In vivo bioluminescence images were taken by Living Image Software (PerkinElmer).<br>H&E staining pictures were taken by LAS V3.8 (Leica).<br>Quantitative PCR were recorded by StepOne software V2.1 (Thermo Fisher).<br>Echocardiography data were taken by Vevo Lab (VisualSonics).<br>TEM images were taken by TEMography (JOEL).<br>Nanoparticle tracking analysis data were recorded by NanoSight NTA (Malvern Panalytical).
Data analysis	Statistical analysis and bar graph was processed or generated by using GraphPad Prism 8.3.

For manuscripts utilizing custom algorithms or software that are central to the research but not yet described in published literature, software must be made available to editors/reviewers. We strongly encourage code deposition in a community repository (e.g. GitHub). See the Nature Research guidelines for submitting code & software for further information.

Data

Policy information about availability of data

All manuscripts must include a data availability statement. This statement should provide the following information, where applicable:

- Accession codes, unique identifiers, or web links for publicly available datasets
- A list of figures that have associated raw data
- A description of any restrictions on data availability

Fig. 1-5 and Supplement Fig.1-4 have associated raw data are available.

Field-specific reporting

Please select the one below that is the best fit for your research. If you are not sure, read the appropriate sections before making your selection.

☒ Life sciences ☐ Behavioural & social sciences ☐ Ecological, evolutionary & environmental sciences

For a reference copy of the document with all sections, see nature.com/documents/nr-reporting-summary-flat.pdf

Life sciences study design

All studies must disclose on these points even when the disclosure is negative.

Sample size	For animal studies, we determined our sample size with 80% confidence level, survival animal population size at 10 (negative control at 8), and 20% margin of error.
Data exclusions	There is no data exclusion in exosome mouse model study.
Replication	All measurements were taken three times. All attempts of replication were successful.
Randomization	Age-, gender- and genetic background-matched animals were randomized by flipping coin.
Blinding	The investigators were blinded to group allocation during datum collection and analysis.

Reporting for specific materials, systems and methods

We require information from authors about some types of materials, experimental systems and methods used in many studies. Here, indicate whether each material, system or method listed is relevant to your study. If you are not sure if a list item applies to your research, read the appropriate section before selecting a response.

Materials & experimental systems

n/a	Involved in the study	
☐	☒	Antibodies
☐	☒	Eukaryotic cell lines
☒	☐	Palaeontology
☐	☒	Animals and other organisms
☒	☐	Human research participants
☒	☐	Clinical data

Methods

n/a	Involved in the study	
☒	☐	ChIP-seq
☒	☐	Flow cytometry
☒	☐	MRI-based neuroimaging

Antibodies

Antibodies used	rabbit anti-CD63 (sc-15363 (H-193), Lot# A2815, Santa Cruz, TX, USA); mouse anti-TSG101 (sc-7964 (C-2), Lot# I2315, Santa Cruz, TX, USA); mouse anti-CD81 (sc-166029 (B-11), Lot#A1217, Santa Cruz, TX, USA); mouse anti-CD9 (sc-13118 (C-4), Lot# K1616, Santa Cruz, TX, USA); mouse anti-GM130 (sc-55590 (H-7), Lot# H2916, Santa Cruz, TX, USA); anti-rabbit IgG HRP-linked (7074S, Lot# 27, Cell Signaling, MA, USA); anti-mouse IgG HRP-linked (7076S, Lot# 33, Cell Signaling, MA, USA).
Validation	Mouse anti-TSG101, mouse anti-CD81, mouse anti-CD9 and mouse anti-GM130 were validated by Western blot showing single band at predicted molecular weight. Rabbit anti-CD63 was validated by Western blot of CD63 and GFP in CD63-GFP fusion protein overexpression cells.

Eukaryotic cell lines

Policy information about cell lines

Cell line source(s)	HEK293 cells were purchased from ATCC.org
Authentication	The cells were authenticated by checking morphology using microscope, growth curve analysis and species verification by isoenzymology.
Mycoplasma contamination	The cell line used in the study was tested negative of mycoplasma contamination
Commonly misidentified lines (See ICLAC register)	N/A

Animals and other organisms

Policy information about studies involving animals; ARRIVE guidelines recommended for reporting animal research

Laboratory animals	8-week to 6-month old male mice with C57/Bl6J background were used in this study.
Wild animals	The study did not involve wild animals.
Field-collected samples	The study did not involve field-collected samples.
Ethics oversight	All animal experiments were conducted in accordance with protocol approved by the Institutional Animal Care and Use Committee of the Texas A&M University Health Science Center-Houston.

Note that full information on the approval of the study protocol must also be provided in the manuscript.
